# Smokeless consumption of medical cannabis pharmacokinetics, safety and feasibility of the CannaHALER© a phase 1a study

**DOI:** 10.1186/s42238-020-00022-4

**Published:** 2020-03-26

**Authors:** Offir Ben-Ishay, Ortal Bar-On, Yoram Kluger

**Affiliations:** Surgical oncology unit, Department of General Surgery, Rambam Health Care Campus and the Bruce and Ruth Rappaport Faculty of Medicine, Technion – Israel Institute of Technology, 8 Ha’aliyah st., 35254 Haifa, Israel

**Keywords:** Inhalation, Medical cannabis, Pharmacokinetics, Δ^9^–THC, 9-CarboxiTHC, Vaporization

## Abstract

**Background:**

Substantial advancements were achieved in the management of postoperative pain, however the need for further improvement remains. This study explores the pharmacokinetics and safety of the CannaHaler, a metered dose inhaler for plant material made by Kite-Systems situated in Tel-Aviv, Israel.

**Methods:**

The study was conducted on 12 healthy adult volunteers divided into four arms (each arm/group holds 3 volunteers) with the evaporated plant material being Alaska strain provided by “Tikun Olam”. This strain is a hybrid of 70% Sativa and 30% Indika strains, consisting of 20–22% THC and 0% CBD. Each arm received a single dose and groups were divided in an ascending dose fashion: Group I-IV receiving 10, 15, 20, 25 mg of THC respectively. The volunteers inhaled a single dose of THC using the CannaHaler, device. Blood samples for Δ^9^ – Tetrahydrocannabinol (THC) and 9-THCCOOH were taken at base line and up to 30 min after dosing. Adverse events were monitored following the inhalation. Pharmacokinetics profile was obtained for each patient in all arms.

**Results:**

Ascending doses of THC produced a linear increase in the maximum concentration 10, 15, 20 and 25 mg of THC. (35.43 ± 5.97, 51.47 ± 13.79, 72.37 ± 15.93, 88.63 ± 14.75 respectively) with the same linear increase in the dimension of the AUC (441.59 ± 88.49, 624 ± 123.56, 698.35 ± 174.98, 971.36 ± 310.4 respectively) both with no change in the time needed to reach such concentration. No adverse events were recorded in all of study subjects. The CannaHaler achieved high C_max_ (35.43–88.63 ng/mL) values and low coefficient of variations (16.64–26.79%) in comparison to both smoking and oral preparations, thus reaching the potential of a pharmaceutical grade device for inhaled substance.

**Conclusions:**

The current study showed that the use of Kite-Systems CannaHaler as a smokeless medical cannabis inhalation device is feasible and efficient. The low coefficient of variation together with the high C_max_ values, suggest the potential use of the CannaHaler device as a pharmaceutical cannabis dosing administrator.

## Introduction

Each year millions of surgeries are performed; most surgical procedures are associated with some degree of postoperative pain. Postoperatively especially in major surgeries, severe pain measured on a 100 mm visual analogue scale (VAS 7–10) is felt at rest and usually resolves within the first 3 days. Pain related to activity will often remain moderate (VAS 4–6) for days or longer. (Brennan 2011; Moiniche et al. 1997) Substantial advancements were achieved in the management of postoperative pain: pain is continuously evaluated and pain management protocols are the standard of care in every modern surgical department, however, the need for further improvement remains.

Many of the commonly used drugs namely opiates and opiate derivates, paracetamol or non-steroidal anti-inflammatory drugs, prove insufficient as a single agent and often are used in combination. Often such combinations prove inadequate for lack of efficacy or multiple side effects. Newer analgesic products are being developed through an in-depth understanding of the neurochemical systems involved in pain processing (Walker et al. 1999; Holdcroft and Patel 2001) including the endocannabinoid system (Mechoulam et al. 1998). Selective cannabinoid agonists have been demonstrated to suppress nociceptive transmission in spinal cord, periaqueductal gray matter, and the thalamus in a dose related manner (Walker et al. 1999). Exogenous cannabinoids have been tested in clinical trials in chronic pain disorders such as visceral pain (Holdcroft et al. 1997), neuropathic pain (Karst et al. 2003; Wade et al. 2003; Attal et al. 2004; Berman et al. 2004; Notcutt et al. 2004; Burstein et al. 2004), and multiple sclerosis (Killestein et al. 2002; Zajicek et al. 2003; Svendsen et al. 2004). Results vary with the clinical setting, possibly because of the diversity of psychological and pathological processes in chronic pain states. The use of selective cannabinoid agonists was never studied for the treatment of acute postoperative pain. Tetrahydro -cannabinolo (THC) and cannabidiol mixtures offers a potentially distinctive role in postoperative pain management for its analgesic qualities, anti-inflammatory effects as well as relief of muscle spasm, reduction of nausea and vomiting, and appetite stimulation (Barden et al. 2004). It may thus support postoperative recovery without adverse effects such as respiratory depression, renal failure, or gastrointestinal ulceration.

Recent advancements in technology allowed the common use of inhalers over the traditional oral or smoking use of cannabis. The inhalers are able to deliver the drug faster with some ability to titrate the doses and better bioavailability than oral administration. The development of the CannaHaler, a cannabis vaporization device aimed at delivering the cannabinoids while avoiding the respiratory hazards of smoking, presents some new promises. The ability to deliver precise doses of THC in a smokeless fashion may play a role in the in-hospital postoperative pain management.

The current study is a phase 1a study that shows the pharmacokinetics, safety and feasibility of the CannaHaler. The study aims to provide a platform for a phase 2 study in patients undergoing abdominal surgery within their postoperative period.

## Materials & methods

### Volunteers

The study was conducted at the surgical oncology unit of the Department of surgery at the Rambam Health Care Campus in Haifa, Israel. Following its approval by the Rambam Health Center Ethics Committee and by the Israeli Ministry of Health. Healthy volunteers were recruited for the study and all gave a written informed consent.

Recruitment for the study was performed through written ads on bulletin boards of the Bruce and Ruth Rapport faculty of Medicine, the Technion institute of technology, Haifa, Israel. Each candidate went through a telephone interview first for the following inclusion and exclusion criteria. Once the candidate was found eligible for the study an on site clinical interview by the medical director of the experiment was conducted (OBI).

Phone interview - Inclusion criteria included: (a) age > 30 and < 70 years; (b) No known medical problems. Exclusion criteria were the presence of (a) significant cardiac or pulmonary disease, (b) history of a psychotic or anxiety disorder, (c) pregnancy, pregnancy attempt or breastfeeding, (d) presence of a neuropathic or non-neuropathic pain disorder, (e) low systolic blood pressure, (> 90 mmHg) (f) diabetes (g) first degree family history of psychotic or anxiety disorder, (h) history of drug addiction, (i) history of drug misuse, (j) concurrent use the following drugs: rifampicin, rifabutin, rarbamazepine, phenobarbital, primidone, (k) using the following plants: *Hypericum perforatum*, troglitazone,

The on site interview repeated the phone interview and included also (a) if applicable, negative urine pregnancy test (β human chorionic gonadotropin pregnancy test), (b) No alcohol consumption up to 12 h prior to the study, (c) abnormal parameters such as heart Rate above 100 BPM, blood pressure, below 90 mmHg (systolic), saturation below 91%, (d) any chronic use of drugs.

### Study protocol

The study has a single-ascending dose design. All participants received a detailed explanation of the study design by the principal investigator. After providing their written informed consent, the study physician obtained patient’s medical history and conducted a thorough physical examination. Detailed instructions on the use of the CannaHaler Inhaler were then provided following by three successful demonstrative inhalations.

The participants were divided into four dose related groups each group included 3 volunteers. After 3 successful training inhalations, each participant inhaled 3 s of a single dose. Dose groups were 10 ± 0.1 mg, 15 ± 0.1 mg, 20 ± 0.1 mg, 25 ± 0.1 mg of THC.

Blood samples were drawn immediately before and at 2, 3, 4, 10, 30 min after inhalation for monitoring of plasma levels of THC and its active metabolite Δ^9^ THCCOOH. The blood was collected in 13 × 75 mm purple-top Vacutainer tubes containing EDTA. Samples were kept on ice and centrifuged within 30 min. Plasma samples were aliquoted into 3.6-mL polypropylene Nunc cryotubes (Thomas Scientific, NJ, USA), stored frozen at − 20 °C, and analyzed within 6 weeks. The cannabinoid analysis was performed at Pactox (Pacific Toxicology Laboratories) Labs by multidimensional gas chromatography mass spectrometry method.

Adverse events were recorded at 5, 15, 30, 60, and 120 min post inhalation, along with those spontaneously reported by the participants. Adverse events were evaluated according to standardized criteria in terms of severity, frequency, duration, and relationship to study drug. Adverse events were graded using the NIH Division of AIDS table for scoring severity of adult adverse experiences (U.S. Department of Health and Human Services, National Institutes of Health, National Institute of Allergy and Infectious Diseases, Division of AIDS 2017).

Blood pressure, pulse rate and oxygen oxymetry were also recorded at baseline, 30, 60, 90 and 120 min post inhalation.

A cognitive test was conducted using the Short-Blessed Test (SBT) (Katzman et al. 1983), a six-item test was used as a diagnostic tool enabling the cognitive status evaluation of the volunteers. Each item was scored and total scores were calculated on the following cut off points: Normal or minimally impaired: 0–8, Moderately impaired: 9–19, Severely impaired: 20–33. The SBT was used prior to the experiment at 30, 60 min after inhalation and at the end of the experiment.

Two hours from the beginning of the trial, examination by the trial physician and pending his decision, participants were discharged after being equipped with a letter to their physician and contact details of the trial manager.

### Study device

**Fig. 1:**
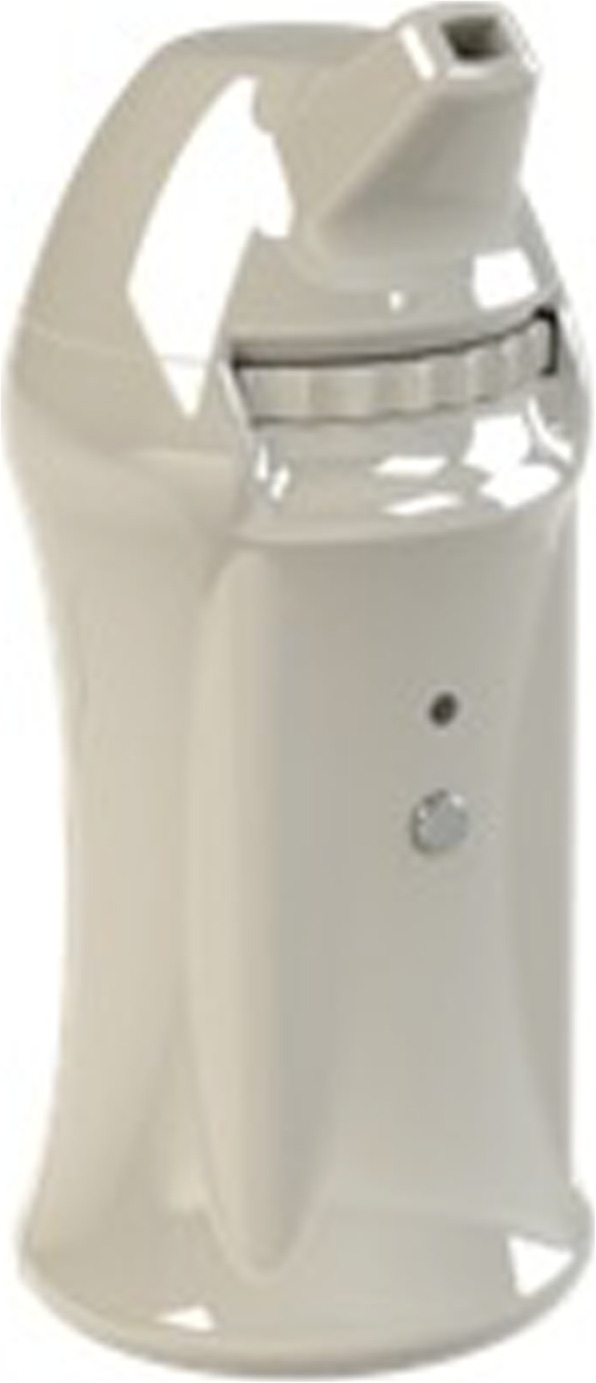
The CannaHaler device

The study device developed by Kite-Systems (Tel-Aviv, Israel) is a battery-operated, small- sized, hand-held thermal metered-dose inhaler, designed to vaporize up to 80 doses of processed cannabis flos, resulting in pulmonary delivery of active ingredients. The Kite-Systems CannaHaler Inhaler consists of a multi-dose cartridge, indication light, and power switch (Fig. 1). The cartridge is preloaded with multiple pre-weighed 10.0 ± 0.1 mg, 15.0 ± 0.1 mg, 20.0 ± 0.1 mg and 25.0 ± 0.1 mg doses of processed cannabis flos. The vaporization process is segmental and is triggered by the volunteer pressing the operating button. The segmental evaporation process is aimed at turning THC_A_ into THC and is done by: (1) stage I – heating the material to 100 °C for 9 s; (2) stage II – heating the material to 150 °C for 9 s; (3) stage III – heating the material to 190 °C to 200 °C (evaporation temperature) for the 3 s of inhalation. The transition to the next inhalation is performed by using a mechanical rotation wheel. The device engages automatic thermal control that ensures a complete, high-efficiency delivery of cannabinoid vapors to the lungs. The Kite-Systems CannaHaler Inhaler can be programmed to accurately deliver specific doses, enabling individualization of THC regimen. The device allows for “single inhalation” dose resolution, instantaneous administration, and requires no preprocessing or any user intervention other than the inhalation itself.

### Study material

The cannabis flowers used in the study were of the Alaska strain provided by “Tikun Olam” (Approved supplier by the Israeli ministry of health). This strain is a hybrid of 70% Sativa and 30% Indika strains. This strain consists of 20–22% THC and 0% CBD. The test samples, were provided by the supplier with relevant test analysis documentation. The processed cannabis flowers that were used in this study were tested for THC by modified gas-chromatography method without derivatization (United Nations Office of Drugs and Crime 2009) resulting in ∆^9^-THC content of 20.08%. The flowers went through a unique processing and loading by Kite-systems, retaining the natural cannabis compounds in their raw form. The study drug was provided preloaded into separate cartridges that delivered the appropriate dosages.

### Study outcomes

The primary outcome of the study was to characterize the inter-individual variability of Δ^9^-THC during the absorption phase. The Secondary outcome included (a) monitoring adverse effects, (b) blood pressure, and heart rate; and (c) monitoring the response of single-ascending dose with the use of the study device. In addition, we have assessed the influence of the use of the trial device on the cognitive status of the user. A Short-Blessed Test cognitive test (Katzman et al. 1983) was used for the purpose of the trial. The test questioner was available in both English and Hebrew.

### Pharmacokinetics and statistical analysis

The cannabinoid analysis was performed at PacTox (Pacific Toxicology Laboratories, Chatsworth, CA 91311, USA) Labs by multidimensional gas chromatography/mass spectrometry method.

The following parameters were directly derived from the study actual experiments and the blood obtained from the study volunteers: (1) each arm Δ^9^ Tetrahydrocannabinol (THC) peak concentration (C_max_ ± SD); (2) Time to reach peak THC concentration (T_max_ ± SD); and (3) 9 THCCOOH. From the Δ^9^ – THC to Time curve a plot was generated, and an area under the curve (AUC) was determined by a linear numerical trapezoidal non-compartmental analysis or the exact method. The results are shown as mean ± SD unless otherwise specified.

### Regulatory considerations

In the current study, we followed the Good Clinical Practice guidelines of the International Conference on Harmonization of Technical Requirements for Registration of Pharmaceuticals for Human Use, the Declaration of Helsinki, concerning medical research in humans (“Ethical Principles for Medical Research”) and all local (Israeli) regulations. A clinical site monitoring was performed by the contract research organizer of the surgical array of Rambam Health Care Campus in Haifa, Israel**.**

## Results

**Fig. 2:**
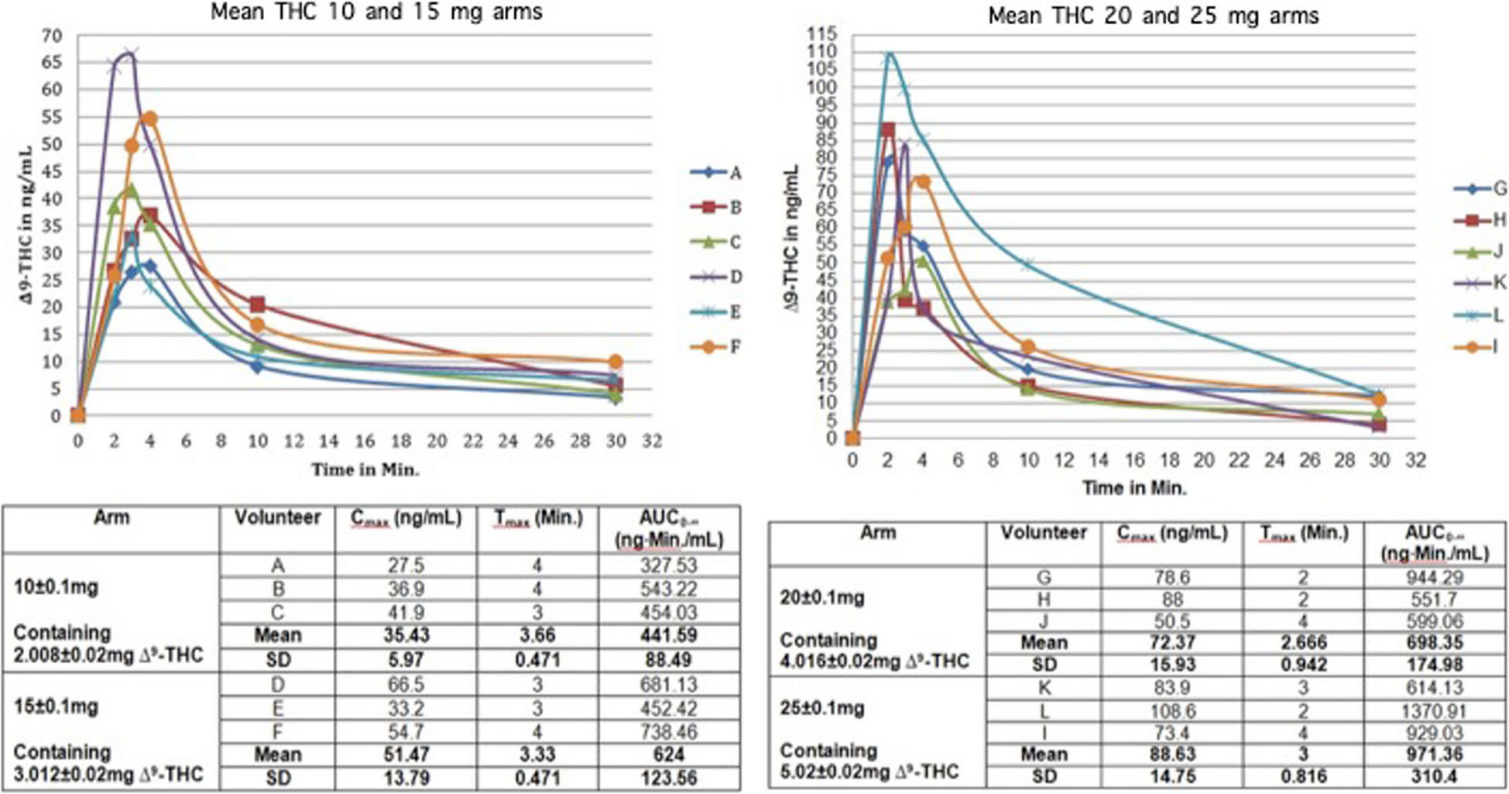
Inter-individual variability of inhaled THC pharmacokinetic parameters. Left pane depicts 10 and 15 mg of THC dosage groups and the right pane 20 and 25 mg and each volunteer was color coded accordingly. The graphic representation shows the maximum concentration (C_max_), area under the curve (AUC) and the time needed to reach this concentration for each volunteer and specific values are showed in the table below

During the current study 12 healthy volunteers were recruited for the experiment. Volunteer’s demographics and morphometrics is depicted in Table 1. The study showed that the use of the CannaHaler with ascending doses of THC produced a linear increase in the maximum concentration of Δ^9^-THC from 35.43 ± 5.97 ng/mL for the 10 mg of THC group to 88.63 ± 14.75 ng/mL for the 25 mg of THC group. The same linear increase was observed in the dimension of the AUC (441.59 ± 88.49 ng-min/mL for the 10 mg THC group to 971.36 ± 310.4 for the 25 mg of THC group) both with no change in the time needed to reach such concentration. Table 2 depicts the mean values of C_max_, AUC and T_max_ of all 4 groups of patients. Figure 2 on the other hand shows a graphic delineation of the inter-individual variability of Δ^9^-THC during the absorption phase accompanied by each patient’s values (A-L). Each line represents a single patient; on the Y-axis we can observe each patient’s C_max_, while the X-axis show the time needed (T_max_) to reach that concentration. The area under the curve was calculate for each patient by the trapezoidal non-compartmental analysis and was expressed in ng-min/mL.

**Table 1:** Baseline characteristics of 12 study volunteers

**Volunteer**	**A**	**B**	**C**	**D**	**E**	**F**	**G**	**H**	**I**	**J**	**K**	**L**
**Dose in mg of THC**	10	10	10	15	15	15	20	20	25	20	25	25
**Gender**	M	F	F	M	M	F	F	M	M	M	M	M
**Age (Years)**	32	32	31	36	51	42	33	34	37	30	30	31
**Weight (Kg)**	67	59.4	60	68.4	69.5	55	53	63	75	72	68	90
**BMI**	26.2	21.8	20.3	22.9	21.5	21.5	22.1	19.4	21.2	22.5	21.5	26.9

Volunteers were assigned a letter alphabetically (A-L) and grouped by dosage. Median age was 32 (30–51) and mean weight and BMI were 61.7 ± 12 and 22.3 ± 2.2 respectively

*BMI* Body mass index

**Table 2:** THC pharmacokinetic parameters after inhalation

	Group I 10 ± 0.1 mg	Group II 15 ± 0.1 mg	Group III 20 ± 0.1 mg	Group IV 25 ± 0.1 mg
Δ^9^-THC C_max_ (ng/mL)	35.43 ± 5.97	51.47 ± 13.79	72.37 ± 15.93	88.63 ± 14.75
AUC_0-∞_ (ng min/mL)	441.59 ± 88.49	624 ± 123.56	698.35 ± 174.98	971.36 ± 310.4
T_max_ (min)	3.666 ± 0.471	3.333 ± 0.471	2.666 ± 0.942	3 ± 0.816

Each THC dosage group concise of four healthy volunteers. Both Δ^9^-THC C_max_ and AUC_0-∞_ measured in plasma of the volunteers and calculated from the same data, are showing a linear increase in mean maximum plasma concentration of THC (C_max_) and area under the curve (AUC) both with no change in the time needed to reach such concentration

*THC C*_*max*_ Tetrahydrocannabinol maximum concentration, *AUC* Area under the curve, *T*_*max*_ time to reach maximum concentration

Mean C_max_ results among the dose groups showed a linear increase but analyzing individual participants, we observed some inter-individual variability. We ran a linear regression analysis to identify variables associated with C_max_ levels. We found that the dose group (p-0.008) and age (p-0.03) were the only factors significantly associated with C_max_ levels while in this cohort of patients weight alone (p-0.7) and BMI (p-0.09) were not.

The temperature sensor and its effective feedback and thermal control algorithm was able to create a low coefficient of variances (CV) expressed in percentage. The CV for the different dosage groups (10,15,20,25 mg of THC) was 16.85, 26.79, 22.01 and 16.64% respectively.

During the time frame of the study no clinically significant change in blood pressure, heart rate or blood oxygen saturation was observed. Table 3 depicts the mean values for each dose group. All volunteers described minimal cognitive impairment. No test subjects showed clinically significant cognitive impairment in any of the dosage groups (Table 4).

**Table 3:** Physiological response to various inhaled doses of THC

	Group I 10 ± 0.1 mg	Group II 15 ± 0.1 mg	Group III 20 ± 0.1 mg	Group IV 25 ± 0.1 mg
SBP (mmHg)	113.4 ± 10.23	114.93 ± 6.88	120.14 ± 28.03	130 ± 16.62
DBP (mmHg)	69.46 ± 7.86	63.85 ± 9.78	71.78 ± 14.95	80.57 ± 8.92
HR	71.66 ± 10.14	79 ± 13.37	68.35 ± 5.83	77.57 ± 17.74
SO_2_ (%)	99.93 ± 0.25	100	99.92 ± 0.26	99.42 ± 0.9

All four THC dosage groups showed no significant change in physiological parameters measured during the study, parameters such as systolic and diastolic blood pressure, heart rate and oxygen saturation

*SBP* systolic Blood pressure, *DBP* Diastolic Blood pressure, *HR* Heart Rate, *SO*_*2*_ Blood Oxygen saturation

**Table 4:** Cognitive function after inhaled THC based on Short-Blessed Test (SBT)

	Group I 10 ± 0.1 mg	Group II 15 ± 0.1 mg	Group III 20 ± 0.1 mg	Group IV 25 ± 0.1 mg
SBT 0 min	0.66 ± 0.94	0	2 ± 1.63	2 ± 2.82
SBT 30 min	2.33 ± 3.29	1.33 ± 1.88	0.66 ± 0.94	0
SBT 120 min	2.66 ± 0.47	3 ± 0.81	0.66 ± 0.94	0

The SBT is a weighted six-item instrument that evaluates cognitive impairment using surrogates for orientation, registration, and attention. Patients in the THC dosage groups showed no change in SBT levels during the time frame of the study

*SBT* Short Blessed test score

During the study experiment, no adverse events of any kind were recorded by staff observation and study physician examination, cognitive and physical examination prior to discharge were normal with scores 0–8 on the Short Blessed Test. The candidates were equipped with a letter to their primary care physician describing the experiment and detailed contacts of the medical director for any adverse events. No such events were reported by the primary care physician.

## Discussion

**Fig. 3:**
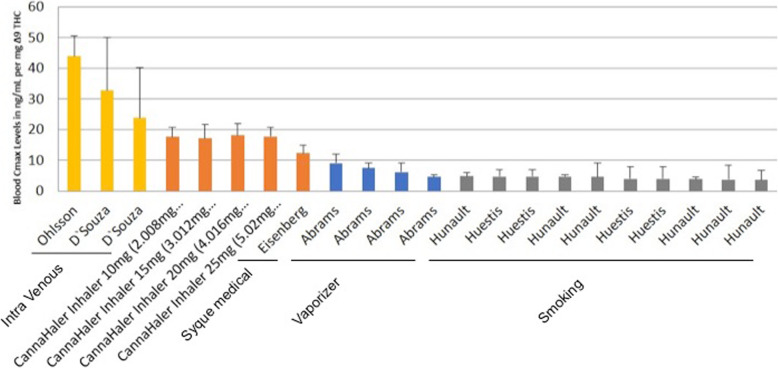
Plasma C_max_ levels per mg of ∆^9^-THC administered by intravenous, inhaler, vaporization and smoking. The CannaHaler yielded the highest increase of THC C_max_ even when compared with the recently published data of the Syqe Inhaler, the Volcano vaporizer and common smoking

**Fig. 4:**
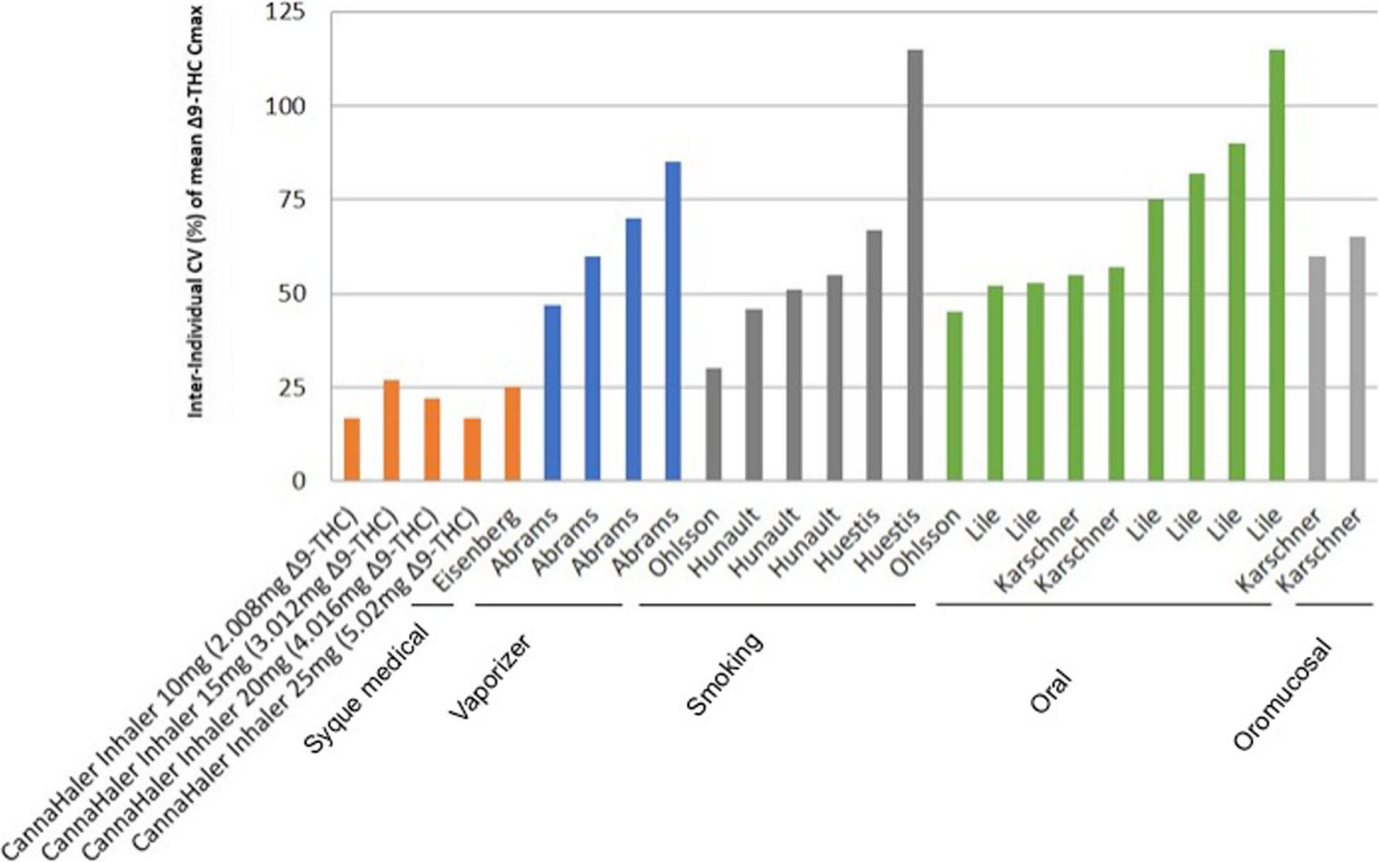
Coefficient of variation CV (%) of THC Cmax by different administration modalities: Inhaler, vaporization, smoking, oral, oromucosal. The CannaHaler showed the lowest CV (%) compared to all the other modalities

The primary outcome of the current study was to determine the pharmacokinetics profile of ∆^9^-THC inhaled by using a heat metered-dose inhaler in an ascending dose fashion. All volunteers inhaled for 3 s ascending doses of THC after a segmented heated evaporation of the flos (whole dried female flower). THC pulmonary absorption is rapid and has a biphasic decline in blood concentration. In comparison, intravenous THC administration yields the highest concentration in the blood i.e. C_max_ in ng/mL of ∆^9^-THC (43.8, 32.8, 23.8) (Ohlsson et al. 1980; D’Souza et al. 2004; Eisenberg and Oginz 2014). Various types of pulmonary delivery methods yield different C_max_ per mg, of THC, Fig. 3 compare the results of the novel CannaHaler with published data in the literature. C_max_ values were collected from each paper separately and compared to the results of the CannaHaler. The CannaHaler yielded the highest increase of THC C_max_ (17–18 ng/mL/mg) even when compared with the recently published data of the Syqe Inhaler (12.3 ng/mL/mg) (Eisenberg and Oginz 2014), the Volcano vaporizer (3.9–9 ng/mL/mg) (Abrams et al. 2007; Abrams et al. 2011) and obviously common smoking (2.9–4.6 ng/mL/mg) (Abrams et al. 2007; Hunault et al. 2008; Hunault et al. 2010). CannaHaler Cmax results are higher by a factor of 3.9–5.8 compared with smoking (Huestis & Hunault) and 1.3–1.46 compared with the syqe inhaler (Eisenberg and Oginz 2014) this is most probably because of the longer inhalation when compared to cigarettes and the transformation to THC from THC_A_ compared to the Syqe inhaler. Because of the CannaHaler inner temperature sensor and its effective feedback and thermal control algorithm, a low coefficient of variances was achieved (16.6–26.8%). Literature CV values reported 32–116% for cigarette (Abrams et al. 2007; Hunault et al. 2008; Huestis et al. 1992), 47–85% For the vaporizer (Abrams et al. 2007; Abrams et al. 2011), 42–115% for oral consumption (Abrams et al. 2007; Karschner et al. 2011; Lile et al. 2013), and 59–67% for oro-mucosal administration (Fig. 4).

The use of the CannaHaler produced no adverse effects and minimal cognitive impairment that was reversible and faded rapidly.

The study is limited by the small sample size, but being a phase 1a study it will serve as a platform for a larger scale phase 2 that will eliminate this limitation and further evaluate the CannaHaler and its possible use in the postoperative pain.

## Conclusions

The current trial demonstrated the pharmacokinetics and feasibility of the CannaHaler medical cannabis inhaler device in ascending doses of up to 25 ± 0.1 mg of processed cannabis flos, containing 5.0082 ± 0.02 mg THC. The low coefficient of variation together with the high C_max_ values suggest the potential use of the CannaHaler device as a pharmaceutical cannabis dosing administration device. These results, combined with the smokeless consumption, precise dosage and the minimal adverse events, produce a good platform for a phase 2 trial for the treatment of acute postoperative pain in patients undergoing abdominal surgery.

## Data Availability

All data generated or analysed during this study are included in this published article and its supplementary information files.

## Abbreviations

THC: Tetrahydrocannabinol
CBD: Cannabidiol
CV: Coefficient of variations
VAS: Viasual Analogue Scale
EDTA: Ethylenediaminetetraacetic acid
